# R. A. McLean

**Published:** 1895-12

**Authors:** 


### OBITUARIES.

THE death of R. A. McLean, D.V.S., of Brooklyn, removes from the veterinary ranks the familiar face of one whose activity for many years was closely associated with every progressive movement in veterinary circles in his own city, State, and country. Graduating from the American Veterinary College with high honors, in 1879, after a long apprenticeship with his father, Dr. L. McLean, still one of the leading veterinarians of the East, he entered upon active practice associated with his father, and was successively connected with the American and New York Veterinary Colleges as instructor in their faculties. He spent many years in United States Government work in the extermination of contagious pleuro-pneumonia, and won for himself an honorable reputation as a thorough diagnostician and aided very much the final triumph of extermination of this disease from our shores. He was connected for many years with the Humane Society of Brooklyn, and also with the State and City Boards of Health. He was one of the organizers of the Long Island Veterinary Society and an ardent worker in the old New York Veterinary Society. As early as 1879 he connected himself with the United States Veterinary Medical Association, and during the years 1887 and 1892 filled many important positions and ardently worked to bring to fruition many of the important changes that have strengthened the organization at home and abroad. He remained at active posts in all his professional connections until the inroads of disease impaired so completely his vital powers, when, after a comparatively short period of confinement to bed, ended his work, at the early age of thirty-nine. Many professional friends and acquaintances attended the funeral ceremony.

